# Non-genomic Actions of Thyroid Hormones Regulate the Growth and Angiogenesis of T Cell Lymphomas

**DOI:** 10.3389/fendo.2019.00063

**Published:** 2019-02-13

**Authors:** Florencia Cayrol, Helena A. Sterle, Maria Celeste Díaz Flaqué, Maria Laura Barreiro Arcos, Graciela A. Cremaschi

**Affiliations:** ^1^Instituto de Investigaciones Biomédicas, Consejo Nacional de Investigaciones Científicas y Técnicas, Facultad de Ciencias Médicas, Pontificia Universidad Católica Argentina, Buenos Aires, Argentina; ^2^Laboratorio de Radioisótopos, Cátedra de Física, Facultad de Farmacia y Bioquímica, Universidad de Buenos Aires, Buenos Aires, Argentina

**Keywords:** VEGF, proliferation, angiogenesis, integrin αvβ3, thyroid hormones, T-cell lymphoma

## Abstract

T-cell lymphomas (TCL) are a heterogeneous group of aggressive clinical lymphoproliferative disorders with considerable clinical, morphological, immunophenotypic, and genetic variation, including ~10–15% of all lymphoid neoplasms. Several evidences indicate an important role of the non-neoplastic microenvironment in promoting both tumor growth and dissemination in T cell malignancies. Thus, dysregulation of integrin expression and activity is associated with TCL survival and proliferation. We found that thyroid hormones acting via the integrin αvβ3 receptor are crucial factors in tumor microenvironment (TME) affecting the pathophysiology of TCL cells. Specifically, TH-activated αvβ3 integrin signaling promoted TCL proliferation and induced and an angiogenic program via the up-regulation of the vascular endothelial growth factor (VEGF). This was observed both on different TCL cell lines representing the different subtypes of human hematological malignancy, and in preclinical models of TCL tumors xenotransplanted in immunodeficient mice as well. Moreover, development of solid tumors by inoculation of murine TCLs in syngeneic hyperthyroid mice, showed increased tumor growth along with increased expression of cell cycle regulators. The genomic or pharmacological inhibition of integrin αvβ3 decreased VEGF production, induced TCL cell death and decreased *in vivo* tumor growth and angiogenesis. Here, we review the non-genomic actions of THs on TCL regulation and their contribution to TCL development and evolution. These actions not only provide novel new insights on the endocrine modulation of TCL, but also provide a potential molecular target for its treatment.

## Introduction

Thyroid hormones (THs), triiodothyronine (T3), and thyroxine (T4), are involved in different biological processes as cell growth, development, differentiation, and the regulation of metabolism and homeostasis (1). The classical mechanism of action of THs is mediated by the binding of T3 to nuclear receptors (TR) that interact with specific responding elements (TREs) in the promoters of target genes. The binding of T3 to TRs promotes a conformational change that induces the exchange of corepressors for coactivators, thus leading to gene transcription on responsive genes (2, 3). THs can also trigger their actions by a non-classical mechanism that does not implicate direct gene transcription regulation by nuclear TRs. These non-genomic actions indirectly modulate gene transcription through the activation of intracellular pathways and other transcription factors (3, 4). Despite many of the non-genomic actions have been demonstrated to be initiated by THs through the activation of a membrane receptor (mTR), they can also be initiated at receptors located in the mitochondria or cytoplasm (5).

In the last years, several studies have identified the integrin αvβ3 as the membrane receptor for THs in normal tissues as blood vessels and heart (5); but also in several types of cancer cells (4, 6–9). Integrin αvβ3 is a member of a large group of heterodimeric transmembrane receptors that regulate cell-cell and cell-extracellular matrix (ECM) interactions and enable cells to respond to their environment (10). Several studies related to cancer have implicated the activity of this group of adhesion receptors in the proliferation, migration, and survival of different types of tumor cells (11). Many aspects of the cellular microenvironment, like the composition and structure of the ECM, the signals generated by growth factors or the stimulation of cytokine secretion are regulated by integrins (12, 13). Particularly, integrin αvβ3 mediates the interaction between the cells and the ECM as a result of its binding to plasmatic and ECM ligands that express the peptide sequence RGD (Arginine–Glycine–Aspartate) (14). Interestingly this integrin is highly expressed in proliferating cells, like malignant cancer cells and cells from the endothelial and vascular smooth muscle (14).

It is well-known that the growth, invasiveness, and dissemination of a tumor are highly associated with angiogenesis. In recent studies, our group demonstrated that the interaction of THs with integrin αvβ3 triggers intracellular pathways in T-cell lymphoma (TCL) cells. This further activates transcription factors, thus stimulating gene transcription and the production of angiogenic factors (15). Therefore, the expression of integrin αvβ3 in tumor cells and their vascular network could explain the proangiogenic and proliferative effects of THs on different cancers, including gliomas (9), breast (4), thyroid (6, 8), and renal cancer (7), among others.

In this review, we will focus on the role of integrin αvβ3 as the membrane receptor for THs and how its activation induces the proliferation and survival of different types of cancer cells. Specifically, we will discuss the influence of THs non-genomic actions through integrin αvβ3 activation on TCL malignant phenotype, and the inhibition of this receptor as a potential clinical target.

## Role of Integrin αvβ3 in Cancer and Angiogenesis

### Integrins and Cancer

Despite integrins were initially described as cell adhesion receptors, current studies highlight the idea that these receptors have essential roles in cancer. In fact, one of the well-known mechanisms of cancer is the abnormal function of integrin receptors (16). Cancer is a complex disease and its progression is deeply related with the dynamically evolving extracellular matrix that regulates many aspects of the tumor and tumor-associated cells (16). Integrin bi-directional signaling is essential to sense, modulate, and respond to changes in extracellular stimuli (17). The signal transduction mediated by these receptors usually occurs through direct or indirect interactions between the cytoplasmic domain of the integrin and intracellular effectors, which occasionally can be supported by the interactions with other cell surface proteins that are associated to integrins (14). For example, it has been reported that caveolin is required for the association between Src-family kinases and β1 integrins; moreover the loss of this association results in the loss of FAK phosphorylation induction and the correct development of focal adhesion sites (18). Tetraspanins, on the other hand, are essential for rapid cell migration mediated by α3β1, α6β1, α6β4, and α7β1 integrins, making these integrin partners potential antimetastatic targets (19). In cancer cells, FAK and Src are two of the best-studied integrin-mediated signaling effectors. Different types of solid tumors, including pancreatic, colon, and breast cancers, show high expression and activation of FAK and Src, thus contributing to the progression and the malignant phenotype of these pathologies (20–22). Inhibition of FAK and Src signaling reduces tumorigenic and metastatic potential of breast cancer cells (23). When integrin-mediated cell adhesion occurs, FAK is activated by autophosphorylation, generating a high-affinity binding site for the SH2 domain of Src. These activated FAK/Src complexes are the link between integrins and the downstream signaling effectors such Rac1 GTPase or the MAPKs (24). The interaction of integrins and their ligands, and the consequent activation of these complexes and the intracellular pathways, can influence cancer cells behavior by increasing cell proliferation, survival, and gene expression; therefore contributing to tumor growth and metastasis (24). All these findings point out the mentioned pathways as potential therapeutic targets in different types of cancer (23, 24).

Most solid tumors are originated from epithelial cells that are conferred with the ability to resist apoptosis, migrate, and disseminate through the epithelial-mesenchymal transition (EMT) (25). This process involves the remodeling of the ECM and changes in the interactions of cells with the ECM (26). Many integrins that are expressed by epithelial cells are retained in the tumor, but their levels and physiologic functions may be altered. Integrins α6β4, α6β1, αvβ5, α2β1, and α3β1, regulate the adhesion of epithelial cells to the basement membrane, however, in tumor cells they might involve and contribute to cell migration, proliferation and survival (11). However, during the differentiation into mesenchymal cells some epithelial integrins are downregulated and the expression of other integrins with key roles in EMT progression and tumor invasiveness are activated (24, 26). For example, the expression of α6β4 integrin is down-regulated during EMT in the mammary gland through the epigenetically silencing of the gene encoding β4 integrin (27). Also in mammary epithelial cells, enhanced expression of integrin αvβ3 is required for TGF-β-induced EMT (28). Likewise, α3β1, α5β1, α1β1, and α2β1 integrins are overexpressed in different stages of EMT (24, 29). Indeed, the expression of many integrin subunits, including α3, α5, α6, αv, β1, β3, and β4 in different types of cancer cells, has been linked to their invasive and metastatic potential (30). The expression of integrins αvβ3, α5β1, and αvβ6 are normally low or undetectable in most adult epithelia but in some tumors their protein levels are overexpressed (11). Elevated αvβ6 integrin levels are associated with fibrosis and cancer in lungs, skin and along the gastrointestinal tract (31). After its activation, α2β1 integrin promotes cell adhesion, proliferation and invasion in liver and lung metastasis (32). In prostate cancer (PCa) integrin α2β1 is overexpressed and its phosphorylation and consequent activation have been associated with the progression of this pathology (33). Also, integrin αvβ3 has been reported to contribute to PCa progression by promoting angiogenesis, survival, and invasion (34, 35). The overexpression of integrin αvβ3 in primary head and neck squamous carcinoma and metastatic lymph nodes was related to lymph node metastasis and worse prognosis (36). In breast cancer, the levels of integrin α6β4 and α*νβ*3 correlate with tumor size, grade and decreased survival (37, 38). The overexpression of integrin αvβ3 is also involved in the switch from a non-tumorigenic state of melanoma to a tumorigenic and invasive one (10) and increased bone metastasis in prostate cancer (39).

It is well-known that integrins are able to synergistically interact with cytokine receptors and growth factors, thus mediating some features of cancer progression as cell migration, invasion, and survival. In the last years, it has been described that integrin N-glycosylation is essential for integrin heterodimerization and interaction with ligands (16, 40, 41). Currently, several published works indicate that N-glycan alterations on integrin subunits influence their affinity for their ligands, thus contributing to the malignant phenotype. These studies propose the targeting of 1,6-GlcNAc structures, sialic acid, and fucose and their related enzymes, in combination with the inhibition of integrins, represent a promising new therapeutic approach (16).

Mainly two therapeutic strategies based on integrin target were developed in the last decades: inhibition of integrin function and the use of integrin expression patterns for drug delivery (42). The direct inhibition of integrin function with synthetic peptides and humanized antibodies, among others, has so far be the main therapeutic strategy in the clinic and until now is the only form of anti-integrin treatment shown to work in patients (43). The antibodies abituzumab, intetumumab, and the small molecule, cilengitide, are the most advanced molecules studied in clinical trials for the treatment of different types of cancer (44). Despite the promising preclinical results observed, poor efficacy was obtained in late-phase clinical trials (16). The problem in translating the preclinical data of anti-integrin therapies to the clinic, especially in cancer, would be related to the poor knowledge of integrin biology. For example, the profile and distribution of many integrins in normal and pathological tissues from cancer patients is somehow hard to achieve as there is a lack of good antibodies for integrin staining in formalin-fixed-paraffin embedded tissues. The use of integrins as biomarkers could improve the efficacy of anti-integrin cancer treatment (44).

In summary, if we improve the skills for the identification of integrins in patient samples and increases our knowledge on other integrin characteristics, as the internalization and intracellular trafficking response in the oncology process, new effective, and safe therapies would be generated.

### Integrins and Tumor Microenvironment

The transformed cells are not capable of generating tumors with metastatic potential by themselves; this process requires a permissive tumor microenvironment (TME) that might be crucial for tumor progression. Recent works have begun to focus more deeply on the study of non-tumor cell components of the stroma and their involvement in the malignant progression (45). The TME include many host cell types, including fibroblasts, endothelial, perivascular, and inflammatory cells, that in some cases can contribute to tumor progression through different processes like angiogenesis, lymphangiogenesis or inflammation. Examples of tumor-associated stromal cells are tumor or cancer-associated fibroblasts (TAFs or CAFs) and tumor-associated macrophages (TAMs) (25, 45, 46). Reciprocal communication between cancer cells and these non-tumoral cells is essential and leads to high proliferation and metastatic capability of the tumor.

Integrins can bidirectionally transduce signals across the cell membrane, (24). The “outside-in” signaling is triggered by chemical or mechanical alterations in the ECM. The interaction of the integrin extracellular head domain with the ECM ligand or divalent cations induces integrin clustering and conformational rearrangements of the cytoplasmic tail that lead to the activation of several signaling pathways that regulate gene transcription and cell shape, survival and migration (47). The “inside-out” signaling, on the other hand, is triggered by a cytoplasmic signal that can alter the integrins' affinity for extracellular ligands (48, 49). These mechanisms are essential for the communication of the cells with their microenvironment and regulate many important biological functions including cell proliferation, survival, and motility. The tumor cells use these same processes to acquire invasive and oncogenic survival properties and to orchestrate changes in the host microenvironment that lead to tumor growth and metastatic dissemination (17).

Additionally to their role in malignant cells, integrins expression on tumor-associated host cells can profoundly influence in the malignant potential of a tumor (17, 50). Integrins are expressed on all the cell types that compose the TME, and modulate functions of both, tumor and stromal cells, that promote the communication between different cell types of the TME, leading to tumor growth and malignant progression (50). For example, integrin α9β1 regulates the signaling that increases tumor growth and lymphatic metastasis via the recruitment of TAFs in breast cancer cells (51). In gastric cancer, C-X-C motif chemokine 12 (CXCL12) derived from CAFs promotes cell invasion by enhancing the clustering of integrin β1 in gastric cancer cells (52). Dr. Cress group demonstrated that the cleavage of integrin α6β1 by the serine protease urokinase plasminogen activator (uPA) induces tumor cell motility, invasion, and metastasis in a xenograft model of PCa cells placed within the living bone matrix (53). The same group described later that TAMs stimulate the production of uPA inside the tumor, resulting in α6β1 integrin cleavage in PCa cells (54).

The capacity of integrins to regulate cell adhesion and migration alone is enough to drive invasion. Tumor cells must break the ECM barriers to metastasize to a distant organ; this process requires not only the degradation and remodeling of ECM, but it can also involve ECM stiffening. For example, in human breast carcinoma, collagen fibers become bundled and align perpendicularly to the basement membrane, thus converting into tracks for cells to migrate (55). Likewise, in pancreatic ductal adenocarcinoma, increased collagen thickness and matricellular fibrosis in response to elevated β1-integrin mechano-transduction was related to a more aggressive pathology (17). ECM degradation and remodeling is carried out by several proteases. It has been shown that integrins can modulate the expression levels and the activity of those proteases, in particular matrix metalloproteinases (MMPs) and the uPA system (56). The ability to regulate matrix organization and remodeling is a critically important function of integrins (24). For example, the interaction between MMPs and integrin β2 is required for leukocyte migration, and the combined participation of MMPs and other integrins is also necessary for tumor metastasis (56).

The levels of MMPs are always elevated in the presence of tumors (57). The expression of MMP gene can be up-regulated by integrin signaling pathways (58). It has been reported in different studies that integrins αv and β1 are able to increase the levels of several MMPs. It was demonstrated that integrin αvβ6 increases the expression levels of MMPs in oral, ovarian and colon cancers (59–61). In oral squamous cell carcinoma (SCC), the increment of integrin αvβ6 expression activates MMP-3, thus promoting oral SCC cell proliferation and metastasis *in vivo* (61); on the other side, integrin β1 promotes invasion and migration of SCC cells vía MMP7 (62). In ovarian cancer cells, high levels of integrin αvβ6 correlate with an augment of the expression and secretion of pro-MMP-2, pro-MMP-9 and high molecular weight uPA, thus increasing ECM degradation (59).

One of the characteristics that is important to consider is the physical location of MMPs because this dictates their biological functions and is critical for tumor progression. The localization of several MMPs in cell membrane through the interaction with integrins has been demonstrated; one example is the binding of MMP-2 to αvβ3 or MMP-9 to αVβ6 (56, 63). MMP-9 expression levels were found to be increased in colon cancer metastasis to liver, and this metalloproteinases co-localized with integrin αVβ6 at the invading border of the tumor (63). Consequently, integrins have a critical role in TME impact on tumor invasion and spreading.

### Integrin αvβ3 and Angiogenesis

Angiogenesis is the formation of new blood vessels from pre-existing ones. Even though it is a fundamental physiological event, in certain situations angiogenesis can also be negative; the formation of new blood vessels contributes to the progression of several pathologies and is crucial in tumor growth and metastasis. Consequently, angiogenesis is essential for the growth, spreading and infiltration of malignant cells within tissues (64). In the beginning, tumors can proliferate and survive by taking advantage of the available vessel of their host and surroundings; nevertheless, malignant cells can become hypoxic if they are too far away from the oxygen and nutrients of those vessels (65). In response to hypoxia tumor cells are able to create new blood vessels to fulfill their metabolic needs.

Tumor angiogenesis depends on ECM disruption, the migratory ability of endothelial cells (ECs) and their adhesion to integrins. As we have already mentioned, integrins are expressed on ECs, lymphatic endothelial cells and pericytes (66) and for this reason, they have been pointed out as important players in cancer angiogenesis (11). They are involved in tumor angiogenesis by interacting with both axis that regulate the maturation and plasticity of the new vessels: the pathway of vascular endothelial growth factor (VEGF) and its receptor (VEGFR) (67) and that of angiopoietins and Tie receptors (ANG-Tie).

Among all integrins, αvβ3 has been thoroughly studied for its localized expression in neovasculature and in aggressive tumors (68). The membrane receptor integrin αvβ3 recognizes ECM proteins expressing the RGD peptide sequence. Despite the expression levels are low in resting endothelial cells and normal organ systems, integrin αvβ3 is highly expressed on activated tumor endothelial cells (11). The latter, makes this integrin an appropriate target for antiangiogenic therapeutics. Moreover, integrin αvβ3 is also express on tumor cells, thus both tumor cells and tumor vasculature can be target by anti-integrin therapy.

It was described that only 20% of integrin αv-null mice survive until birth, and that 100% die within the 1st day of birth (69). These mice develop intracerebral hemorrhage due to the defective interactions between blood vessels and brain parenchymal cells (70). On the other side, the β3 integrin-null mice can survive and apparently develop a normal vascular network (71). Furthermore, no integrin β3 protein levels are detected in quiescent blood vessels, but its expression increases during sprouting angiogenesis (72).

One of the roles of integrin αvβ3 during angiogenesis is to bind and activate MMP-2 on new blood vessels to disrupt ECM and facilitate tumor cell migration and infiltration (64). A cooperative action between activated integrin αvβ3 in tumor cells and platelets, that promotes extravasation and metastasis, has also been reported (73). Integrin αvβ3 also participates in the angiogenic switch. This process is referred the time during tumor progression where the balance between pro- and anti-angiogenic factors tilts toward a pro-angiogenic outcome, resulting in the transition from not vascularized hyperplasia to a vascularized tumor and malignant tumor progression (74). In this sense, it was described that the inhibition of tumor-associated αvβ3 integrin regulates the angiogenic switch in melanoma cells leading to reduced melanoma growth and angiogenesis *in vivo* (74).

In 2004, Davis et al. have shown that THs can induce angiogenesis through a cell surface receptor using a chick chorioallantoic membrane (CAM) model (75). In 2005, Bergh et al. have demonstrated that the membrane receptor for THs is near the RGD binding site of the integrin αvβ3 (76). Additionally, we found that the activation of integrin αvβ3 by THs mediates angiogenesis in malignant T cells (15). A number of *in vitro* and *in vivo* studies have supported a role for THs in the proliferation of tumor cells (75, 77–79) and as proangiogenic factor in many types of cancer (15, 75, 76, 80). These properties may be relevant to tumor biology and we will discuss them later in this review.

All the mentioned findings highlight integrin αvβ3 as a fundamental tumor angiogenic promotor. Antagonists of αvβ3 integrin were developed and some proved to be very successful antiangiogenic agents both *in vitro* and in preclinical angiogenesis assays *in vivo*. In accordance, integrin αvβ3 antagonists could inhibit tumor growth in several cancer animal models of human breast cancer (81) and glioblastomas (82). Cilengitide, a specific inhibitor of integrin αvβ3, was able to decrease tumor growth in two different angiogenic and invasive glioblastoma models, by decreasing the diameter of tumor vessels thus reducing the infiltration of cells around the tumor center (83). Associated with its function as membrane receptor for THs actions, the effects of the deaminated analog of L-thyroxine, Tetraiodothyroacetic acid (TETRAC) and its nanoparticulate formulation have been reported as antithyroid agents at the integrin (84).

## Thyroid Hormone Non-Genomic Actions in T cell Lymphomas

### THs Effects on T Cell Lymphoma Growth and Proliferation

As we have already mentioned, THs are critical for many processes like cell growth, differentiation, metabolism, and homeostasis maintenance (1). The classical effects of THs are initiated when T3 binds to their nuclear receptors (TRs) that interact with specific responding elements (TREs) in the promoters of target genes. The conformational change promoted by the binding of T3 to TRs induces the exchange of corepressors for coactivators, thus leading to gene transcription on responsive genes (2, 3). TRs are encoded by two different genes: the THRA located in chromosome 17, and the THRB located in chromosome 3, codifying for the TRα and TRβ proteins, respectively (2, 3). The expression of these isoforms differs during the embryonic development and in adult tissues (1). Mutations of TRs have been detected in several cancers, such as erythroleukemia and liver, kidney and thyroid cancers (13). These mutations have been suggested to be a selective advantage for malignant transformation (85). Thus, the mutation (86, 87) or aberrant expression (88) of TRs has been demonstrated in several cancer cell lines. Also, biopsies of patients with gastrointestinal tumors showed increased levels of TRα1 that correlate with Wnt pathway activation and tumor proliferation (89).

Several clinical studies show controversial results related to THs status and cancer. On one side, some studies show that hyperthyroidism might be a risk factor for the development and progress of different types of tumors like breast, thyroid and prostate cancers (85, 90, 91), while hypothyroidism could favor the clinical outcome of cancer patients (92, 93). However, hypothyroidism was associated with an increased risk of colorectal cancer and hepatocellular carcinoma, that would be explained by the increased generation of reactive oxidative species associated with lipid peroxidation, that result in chronic inflammation and DNA damage leading to neoplastic transformation (94, 95). The association between THs and cancer is now better understood following the discovery of the αvβ3 integrin plasma membrane receptor for T4 and T3 (see below).

In the last decade several studies reported the proliferative effect that physiological concentrations of T3 and T4 have on different cancer cell lines, such as glioma, papillary, and follicular thyroid carcinoma, lung carcinoma and breast adenocarcinoma, among others (26, 77, 78, 96). These actions induce the activation of intracellular signaling pathways and transcription factors that increase cell proliferation.

In this sense, our group has investigated the effect of genomic and non-genomic actions of THs on normal T lymphocytes (97, 98) and in TCL cell (15, 79, 99–103) proliferation and survival. We found that TH induced cell proliferation of murine TCL cells by triggering a non-genomic intracellular signaling that involves the activity of PKCζ that leads to ERK 1/2 and NF-κB activation and the increase of transcriptional levels of TRs and the inducible nitric oxide synthase (99). We have also found that THs can regulate the balance between proliferation and apoptosis of TCL cells both *in vitro* and in *in vivo* assays (79, 100). Additionally, we studied how the thyroid status modulates the *in vivo* growth of EL4 TCL cells and how the antitumor immune response is affected in euthyroid, hypothyroid, and hyperthyroid mice. The appearance of palpable solid tumors was earlier in hyperthyroid animals, which also developed tumors with a higher growth rate and an increased volume when compared with tumors in euthyroid controls or hypothyroid mice (79). In addition, the larger tumor size in hyperthyroid mice was accompanied by higher expression levels of the proliferating cell nuclear antigen and cell cycle regulators; and with an increase of intratumoral and peritumoral vasculogenesis (79).

**Figure 1:**
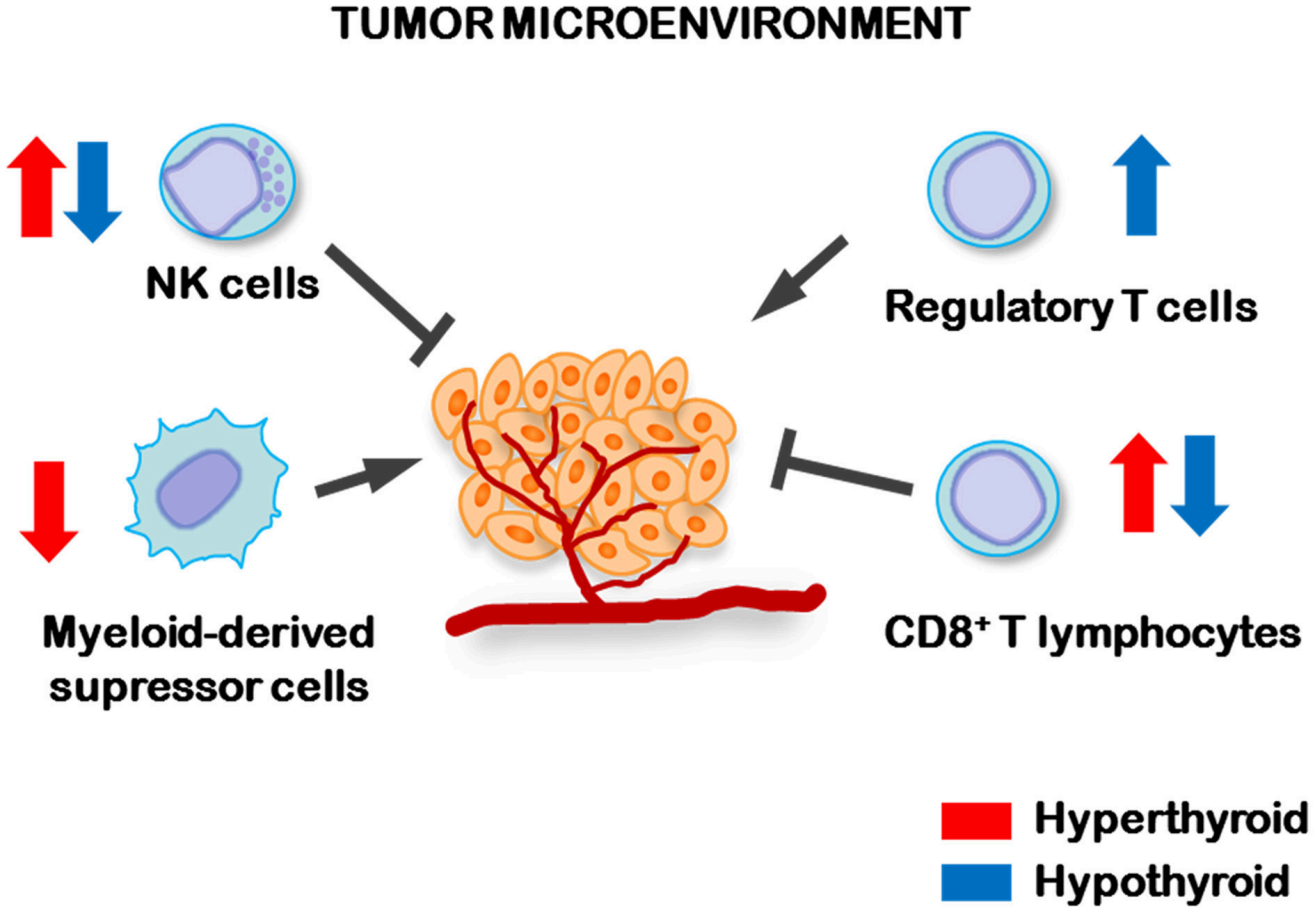
Regulation of antitumor immune cells by circulating levels of THs. In hyperthyroid conditions (blue arrows) increased number and activity of NK and cytotoxic CD8+ lymphocytes, while decreased number of myeloid-derived suppressor cells, were found in the spleens of TCL tumor-bearing mice. However, hypothyroid tumor-bearing animals displayed higher numbers of T regulatory lymphocytes (Treg) in tumor-draining lymph nodes and lower number and activity of splenic NK and CD8+ lymphocytes than control, indicating that the hypothyroid status favors the dissemination of TCL cells.

Despite TCL tumor growth was not significantly different between hypothyroid and euthyroid mice, hypothyroid animals showed a higher frequency of metastases (102). This was associated to an increased percentage of regulatory T (Treg) cells in their tumor draining lymph nodes, a decrease number and activity of splenic NK cells and a decreased number of splenic myeloid-derived suppressor cells (MDSCs) when compared to control euthyroid tumor-bearing mice (102) (Figure 1). Also, tumor-bearing hyperthyroid mice displayed the lowest metastatic dissemination. This was related with an increased systemic antitumor immunity in hyperthyroid mice, reflected by the low number of MDSCs and increased number and activity of both NK and CD8+ cytotoxic T lymphocytes (Figure 1), thus strengthening the fact that low levels of circulating THs are related to TCL spreading and metastatic dissemination. These results highlight the importance of monitoring the thyroid status in patients with TCL.

### Integrin αvβ3 as the Thyroid Hormone Membrane Receptor in TCL Cells

As we have already mentioned, both T3 and T4, play important roles in regulating the proliferation of several cancer cell types. Their metabolic, developmental and growth effects in normal tissues are mediated primarily by TRs (104), while their surface receptors are involved in the modulation of angiogenesis. Bergh et al. (76) found that physiological concentrations of T4 activate the MAPK pathway in CV-1 cells that lack nuclear TRs, but express the mTR integrin αvβ3. The MAPK-mediated proangiogenic action of T4 was inhibited by TETRAC, RGD peptide, and anti-αvβ3 antibodies (76). These results indicated not only that the surface receptor for THs is on the integrin αvβ3, but also that the binding site for the hormone is either at or near the RGD recognition site. High affinity-binding of radiolabeled hormone to the purified integrin was also demonstrated, and for a complete identification of the mTR, knockdown of integrin αvβ3 by small interfering RNA (siRNA) against both monomers was shown to abrogate the transduction of the THs signaling into MAPK activation (105).

Many laboratories reported the involvement of ERK1/2, Src kinase, and PI3-kinase in the non-genomic mechanisms of THs (106, 107). Studies performed in a glioblastoma cell line showed that not only T3, but also T4 activate the ERK1/2 pathway leading to cell proliferation (26). These results point out a difference between mTR and TRs, the latest is activated with high affinity only by T3, while integrin αvβ3 can bind both T3 and T4.

Studies of the kinetics of thyroid hormone binding performed with crystallographic and mathematical modeling (108, 109) found that THs binding site on integrin αvβ3 has no homology to nuclear TR and contain two binding domains. One domain, namely S1, recognizes exclusively T3 and activates PI3K via Src kinase. The S2 domain regulates MAPK1 and MAPK2 and binds both T4 and T3, however the affinity for T4 is higher than the S1 or S2 sites have for T3 (5). At physiological free hormone concentrations T4 is maximally active at the S2 site on integrin αvβ3, however significantly higher than physiological levels of free T3 are required to induce proliferative activity via this receptor (5).

The identification of αvβ3 integrin as the mTR provides the molecular basis to many actions of TH at cancer cells. THs can influence cell proliferation, survival and angiogenesis in different cancer cells via integrin αvβ3 (110– 112). Thus, myeloma cell adhesion to fibronectin is increased by T3 and T4 which induces αvβ3 clustering. In addition, THs induce MMP-9 expression and activation via integrin αvβ3 and MAPK induction, suggesting a role for TH-mediated activation of integrin αvβ3 in myeloma migration and progression (110). THs also promote the proliferation of ovarian cancer cells via integrin αvβ3 that activates extracellular regulated kinase (ERK1/2) (112). In breast cancer cells, THs regulate cell migration via integrin αvβ3 that activates SRC/FAK/PI3-K pathway (111).

### Integrin αvβ3 in the Malignant Phenotype of T Cell Lymphomas

T cell lymphomas (TCL) are a broad group of aggressive lymphoproliferative disorders with significant variation clinical, immunophenotypic, and genetic features. This group of hematologic disorders that is characterized by a clonal growth of T cells at different stages of maturation represents ~10–15% of Non-Hodking lymphomas (113, 114). The last World Health Organization classification has divided this group of hematopoietic malignancies according to its predominant presentation: leukemic (disseminated), nodal, extranodal, or cutaneous (115). The most frequent subtypes include peripheral T cell lymphoma not otherwise specified (PTCL-NOS), anaplastic large cell lymphoma (ALCL) and angioimmunoblastic T cell lymphoma (AITL) (116, 117). Although cutaneous T cell lymphomas (CTCL) are less frequent, is important to note that the skin is the second location in frequency of extranodal primary lymphomas (118). As in other neoplastic disorders, TCL are exposed to a complex environment that comprises among others, growth factors, cytokines, and hormones that are produced by either lymphoma cells or normal cells present in the surrounding or distal tumor microenvironment (119, 120). As we already mentioned, we have demonstrated that one of those factors are THs (15, 79, 99, 100, 103).

Studies of our group demonstrated that both, genomic and non-genomic actions triggered by THs increase cell proliferation of human and murine TCL lines. Moreover, these results described the contribution of the mTR, the integrin αvβ3, in the non-genomic actions of THs in TCL cells (15, 99, 103). The signaling induced by THs through the mTR in murine TCL cells includes the rapid translocation of the ζ isoform of PKC to the cell membrane (99, 103), ERK 1/2 phosphorylation and the activation of the transcription factor NF-κB (15, 99), all molecular processes that are essential for the proliferation and survival of TCL cells.

Recently, we have also demonstrated that integrin αvβ3 is the mTR in human TCL cells. Both T3 and T4 were able to induce *in vitro* the proliferation of tumor, but not normal T lymphocytes (99, 103), being the presence of physiological concentrations of both hormones the most effective to trigger the growth of human TCL cell lines (15). Thus, in a panel of 9 human TCL cell lines, representing the different subtypes of the disease, we showed that the proliferative actions triggered by THs were mediated by the activation of integrin αvβ3. This effect was blocked when the mRNA levels of the integrin αv, β3, or both were downregulated using siRNA (15). Additionally, we have evidenced that these effects were accompanied by the regulation of cell cycle markers. According to this, it has been reported in breast cancer cells that TETRAC inhibits the effects of THs on the integrin αvβ3 leading to an increment in the expression of proapoptotic genes, demonstrating that THs non-genomic actions are required for the survival of these cells (4, 121).

We identified the genetic programs activated by THs through their actions on integrin αvβ3 in TCL cells. To this aim we performed RNA sequencing techniques on TCL cells and analyzed results using bioinformatic tools. We found that genes involved in protein translation, lymphocyte proliferation/differentiation, DNA replication and angiogenesis were mobilized by THs through the mTR activation. Remarkably, we found that the intracellular pathways activated by THs through the mTR significantly induced the transcriptional levels VEGFA and VEGFB genes. This induction was abrogated by siRNA against integrin αvβ3 in TCL cells either from immature or mature origins; and dependent on the activation of the transcription factor NF-κB (15). Importantly, when we performed these experiments in the presence of vitronectin, the “natural” ligand of integrin αvβ3, we found that the pathways triggered by THs are different.

**Figure 2:**
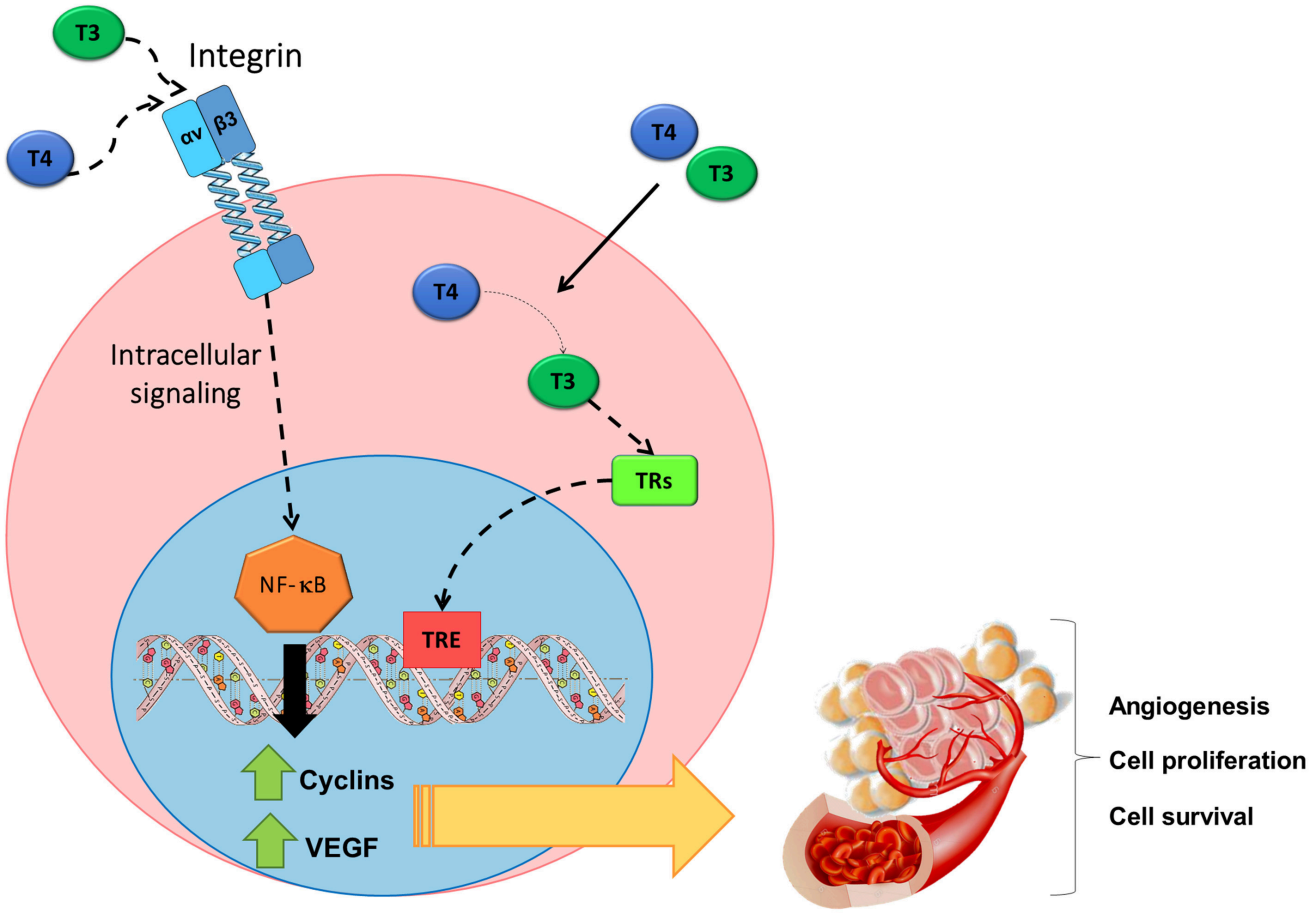
Non-genomic action of THs initiated at the surface receptor of TCL cells on the integrin αvβ3. THs induce signaling pathways triggered after binding to integrin αvβ3 include the activation of NF-κB, thus leading to the production of angiogenic factors such as VEGF and to cell proliferation, cell survival and angiogenesis. Figure adapted from Cremaschi et al. (122) with permission from Elsevier.

It is important to note that it was also evident an association between integrin αvβ3 and VEGF expression in samples from patients with PTCL. By bioinformatic analysis of PTCL tissue microarrays we found a positive correlation between the transcriptional levels of integrin αv or β3 and those of VEGFA or VEGFB. We also verified that the induction of VEGF production in TCL that is regulated by THs functions in a paracrine or autocrine manner. The induction of VEGF production mediated by THs increased the migration of human endothelial cells, and tumor cell proliferation. Moreover, the blocking antibody against VEGF, bevacizumab, abrogated all the mentioned effects. We also found that the proliferative action triggered by THs on TCL cells was impaired by the inhibitor of VEGF receptor, Axitinib, (15, 122). All these findings are resumed in Figure 2. In sum, we found that the transcriptional programs initiated by THs, through the activation of integrin αvβ3, stimulate cell proliferation and favor cell survival of TCL, thus, contributing to their malignant phenotype. Furthermore, they also lead to the production and release of angiogenic factors, thus favoring tumor dissemination.

### Inhibition of Integrin αvβ3 Receptor for TCL Treatment

As we have already mentioned, integrin αvβ3 is highly expressed on activated tumor endothelial cells, but not on resting endothelial cells and normal organ systems (11). In addition, this membrane receptor is also highly expressed on tumor cells. This characteristic makes integrin αvβ3 an attractive target for both tumor cells and tumor vasculature.

Based on the proliferative and proangiogenic roles of THs mediated by the integrin αvβ3 in TCL cells, we used preclinical models to analyze whether these pathways could be capitalized for the treatment of patients with TCL. We performed xenografts of human TCL in NOD-SCID immunodeficient mice and we evaluated the effect of integrin αvβ3 inhibition on tumor growth. The negative regulation of the integrins αv or β3 in TCL cells by siRNA reduced the tumor volume and decreased the protein levels of VEGF and the blood vessel area in TCL tumors (15, 122). This suggests a decrease in the angiogenic potential of tumors derived from cells that do not express the integrin αvβ3. We then wondered whether integrin αvβ3 actions on lymphoma cells could be therapeutically capitalized for the treatment of TCL patients; and considering that PTCL-NOS is the most frequent subtype, we developed a xenograft model of human PTCL-NOS cells into SCID mice and evaluated the action of the selective inhibitor of integrin αvβ3 cilengitide. We found that cilengitide treatment reduced tumor volume by decreasing NF-κB pathway activation and the microvascular lumen size, while increasing tumor apoptosis (15). Moreover, similar effects were found in mice bearing ALCL patient-derived tumors (PDX) xenografts (15, 122). It is important to note that in mice treated with cilengitide no toxic effects were observed. These results highlight the importance of these mechanisms for the development of a more effective and less toxic therapy for patients who suffer these pathologies.

Cilengitide was the first integrin antagonist evaluated in clinical phase I and II trials for the treatment of glioblastoma and several other tumor types (123–125). No encouraging results were found in patients with glioblastoma when using cilengitide as a single agent. Some reasons for the unexpected clinical low efficacy in glioblastoma could be related to the fast off-rate of cilengitide from its targets, the rapid plasma clearance, or the poor perfusion of the brain tumor environment (43). However, it is important to note that a beneficial therapeutic action was found when administered in association with standard radiotherapy or chemotherapy (125, 126), and this was also found in other type of tumors (127, 128).

There is not much information on the role of THs and its action on integrin αvβ3 in other hematologic malignancies; however it was shown that this integrin enhance the proliferation of acute myeloid leukemia (AML) cells (129) and it is required for AML cell survival (130). Furthermore, integrin αvβ3 expressed on the worst prognostic AML cells mediates the interaction with stroma cell-derived ligands in the bone marrow niche, thus triggering a signaling cascade that is critical for the proliferation of AML cells (131). Activated integrin αvβ3/β-catenin signaling pathway in tumor microenvironment decreased the sensitivity of AML cells to tyrosine kinase inhibitor sorafenib, as well (132). Thus, inhibition of this integrin signaling pathway would also be of potential therapeutic impact in AML.

## Concluding Remarks

Integrins are crucial mediators for the survival and migration of tumor cells, not only by acting directly on these cells, but also through the influence they exert on the cells of the microenvironment that surround the tumor. Due to the central role that integrins play in tumor angiogenesis and metastasis, they have become promising targets for the treatment of different types of aggressive cancers.

In this sense, integrin αvβ3 has a crucial role in inducing tumor cell migration and metastasis to distant organs. Moreover, being the membrane receptor for thyroid hormone non-genomic actions, integrin αvβ3 triggers intracellular pathways leading to TCL proliferation and survival and to tumor growth and vascularization via the production of angiogenic factors. The selective inhibition of the integrin αvβ3 in different subtypes of TCL results in the decrease of cell proliferation, tumor growth and impaired angiogenesis. The lack or low expression of integrin αvβ3 in non-active endothelial cells and in normal lymphoid cells, important actors in antitumor immune response, offers a rationale and attractive target for TCL treatment.

Moreover, integrin αvβ3 may be an attractive therapeutic tool for other neoplastic diseases. In fact, in patients with advanced solid tumors, as breast, ovary, and pancreas cancers, the benefit of medical induction of euthyroid hypothyroxinemia was demonstrated (133–136). These studies were based on the fact that integrin αvβ3 is overexpressed in these types of tumors, and, by reducing T4 levels, the cancer cell proliferation and survival and the tumor-related angiogenesis can be reduced, without affecting other important metabolic processes that are mainly regulated by T3 levels.

## Author Contributions

FC: conception and design, acquisition of data, writing/drafting manuscript, revising for important content, final approval of version to be published agreement for accountability of published material; HAS: writing/drafting manuscript, revising for important content, final approval of version to be published; agreement for accountability of published material; MD: revising for important content, final approval of version to be published; agreement for accountability of published material; MB: revising for important content, final approval of version to be published; GC: conception and design, writing/drafting manuscript, revising for important content, final approval of version to be published; agreement for accountability of published material.

### Conflict of Interest Statement

The authors declare that the research was conducted in the absence of any commercial or financial relationships that could be construed as a potential conflict of interest.
